# Detection of *Clostridium perfringens* alpha toxin gene in lambs by loop mediated isothermal amplification

**DOI:** 10.14202/vetworld.2016.60-64

**Published:** 2016-01-20

**Authors:** B. Radhika, N. Vinod Kumar, D. Sreenivasulu

**Affiliations:** 1 State Level Diagnostic Laboratory, College of Veterinary Science, Tirupathi, Andhra Pradesh, India; 2 Department of Veterinary Microbiology, College of Veterinary Science, Tirupathi, Andhra Pradesh, India

**Keywords:** *Clostridium perfringens*, enterotoxemia, lambs, loop mediated isothermal amplification

## Abstract

**Aim:**

The loop mediated isothermal amplification (LAMP) was standardized for rapid detection of *Clostridium perfringens*.

**Materials and Methods:**

A total of 120 fecal samples were collected from enterotoxemia suspected lambs were used for screening of *C. perfringens cpa* gene by LAMP. The specificity of the LAMP amplified products was tested by digesting with restriction enzyme *XmnI* for alpha toxin gene.

**Results:**

Out of 120 samples screened 112 (93.3%) samples were positive by both LAMP and polymerase chain reaction (PCR) for detection of *cpa* gene which indicated the equal sensitivity of both the tests. The enzyme produced single cut in 162 base pair amplified product of alpha toxin gene at 81 base pair resulting in a single band in gel electrophoresis.

**Conclusion:**

Both LAMP and PCR for detection of *cpa* gene indicated the equal sensitivity of both the tests. Standardization of LAMP reaction for amplification of epsilon and beta toxin genes will help to identify the *C. perfringens* toxin types from the clinical samples. The test could be a suitable alternative to the PCR in detection of toxin types without the help of sophisticated machinery like thermal cycler. Considering its simplicity in operation and high sensitivity, there is the potential use of this technique in clinical diagnosis and surveillance of infectious diseases.

## Introduction

The *Clostridium perfringens* is a Gram-positive anaerobic, spore forming, rod-shaped bacteria that commonly found in the gastrointestinal tract of both humans and animals [1]. The organism is classified into five toxinotypes (A to E) based on the production of four major toxins, namely alpha (*cpa*), beta (*cpb*), epsilon (*etx*), and iota (*itx*). *C. perfringens* Type A is implicated in ovine and caprine enterotoxemia in some parts of the world. The *cpb2* producing *C. perfringens* Type A has also been linked to disease in several animal species, including sheep and goats and poultry [2,3].

Various polymerase chain reaction (PCR) protocols including multiplex PCR assays have been established to genotype *C. perfringens* isolates [4,5]. PCR got several intrinsic disadvantages, such as requirement of thermal cycling, time-consuming, post-PCR analysis, and high-risk for cross-contamination [6]. Loop mediated isothermal amplification (LAMP) is a novel nucleic acid amplification which relies on auto-cycling strand displacement DNA synthesis performed by the *Bst*DNA polymerase large fragment [7-10].

Taking above factors into consideration, attempts were made in the present study to develop LAMP with ease of operation for amplification of *cpa* gene of *C. perfringens*.

## Materials and Methods

### Reference strain

*C. perfringens* Type D reference strain (Microbial Type Culture Collection [MTCC]No.49) was obtained from MTCC, Chandigarh was used for standardization of LAMP and as positive control for multiplex PCR reactions. The DNA was extracted from 24 h culture of *C. perfringens* reference strains by phenol-chloroform-isoamylalcohol method [11].

### Collection and processing of samples

A total of 120 dung samples were collected from enterotoxemia suspected lambs of 1-3 months age group. The samples were collected aseptically with sterile swabs and transferred to the lab under ice within 1-2 h. Samples were suspended in sterile phosphate buffer saline and kept in the water bath at 80°C for 5-10 min to eliminate enteric contaminant. The supernatant was inoculated into Robertson’s cooked meat medium and incubated anaerobically with anaerobic gas pack (BD, USA) in anaerobic gas jar at 37°C for 24 h. The DNA was extracted from the 24 h broth culture by boiling method and the sample was preserved at −20°C.

### Primer design

**Figure-1:**
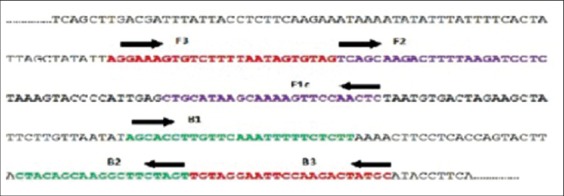
Loop mediated isothermal amplification primers location Clostridium perfringens plc gene.

Toxin gene specific LAMP primers were designed on the basis of the published sequence of *cpa* gene of *C. perfringens* (GenBank accession number, JX091649) with the LAMP primer design software program Primer Explorer V4, from Ekin Chemical Company, Japan (http://primerexplorer.jp/elamp3.0.0/index.html). A set of four AT-rich primers comprising two outer and two inner primers were designed. The two outer primers were known as the forward outer primer (F3) and the backward outer primer (B3), which helps in strand displacement. The inner primers were known as the forward inner primer (FIP) and the backward inner primer (BIP), respectively. FIP contains F1C (complementary to F1), a TTTT spacer and the F2 sequence. BIP contains the B1C sequence (complementary to B1), a TTTT spacer and B2 sequence. The primer details and location were given in Table-1 and Figure-1. Theprimers were procured from Eurofins Genomics India Pvt. Ltd., Bengaluru.

**Table-1:** Primers used in LAMP for *cpa* gene amplification.

Oligoname	5’position	3’position	Length	Tm	GC content (%)	Sequence
F3	351	375	25	56.45	32	AGGAAAGTGTCTTTTAATAGTGTAG
B3	537	558	22	57.30	41	GCATAGTCTTGGAATTCCTACA
FIP			47			GAGTTGGAACTTTTGCTTATGCAG-
						TCAGCAAGACTTTTAAGATCCTC
BIP			44			AGCACCTTGTTCAAATTTTTCTCTT-
						ACTAGAAGCCTTGCTGTAG
F2	376	398	23	57.94	39	TCAGCAAGACTTTTAAGATCCTC
F1c	416	439	24	60.59	42	GAGTTGGAACTTTTGCTTATGCAG
B2	518	536	19	55.49	47	ACTAGAAGCCTTGCTGTAG
B1c	471	495	25	60.12	32	AGCACCTTGTTCAAATTTTTCTCTT

LAMP=Loop mediated isothermal amplification, FIP=Forward inner primer, BIP=Backward inner primer

### Standardization of LAMP reaction

The enzyme *Bsm* DNA polymerase was procured from Thermo Scientific, Inc. The LAMP was standardized to determine optimum concentrations of primers, enzyme, temperature and time combinations for amplification of *cpa* gene. Total volume made up to 25 µl using nuclease free water with MgCl_2_ (25 mM)-1 µl; *Bsm* buffer (×10)-2.5 µl; dNTP mix (10 mM)-3.5 µl; *Bsm* DNA polymerase (8 U/µl) – 1 µl; temple (target DNA) – 2 µl; F3 (10 pmol/µl) – 1 µl; B3 (10 pmol/µl) – 1 µl; FIPα (40 pmol/µl) - 4 µl; BIP (40 pmol/µl) - 4 µl; nuclease-free water – 5 µl.

### LAMP amplification

The LAMP amplification was done in hot water bath with thermostat by the following steps with negative controls added with nuclease free water in place of template DNA.

In the initial reaction nuclease free water, *Bsm* buffer (×10), dNTP mix and template were added into ependorff tube with initial denaturation at 95°C for 5 min. After chilling the samples for 30 s to 1 min, outer primers, inner primers and enzyme were added. LAMP reaction was allowed for 60 min at 55°C followed by enzyme inactivation step for 10 min at 80°C. The amplified LAMP products were stored at −20°C.

LAMP amplification reaction was carried out in hot water bath at 55°C for 60 min. Enzyme inactivation was carried out after completion of reaction at 80°C for 10 min. The amplified LAMP products were stored at−20°C.

### Electrophoresis of LAMP products

LAMP products were subjected to agarose gel electrophoresis with 3.0% agarose in tris-borate-ethylenediaminetetraacetic acid buffer with ethidium bromide at 5 µg/ml in genie submerged gel apparatus at 35 mA and amplified products were visualized under ultraviolet (UV) transilluminator and photographed with gel documentation system (Alpha Innotech, Alphaimager, HP).

### Testing the specificity of LAMP for cpa using restriction enzyme

To confirm that the amplification product had corresponding DNA sequences, the amplified product was digested with restriction enzyme *Xmnl* (New England Biolabs) with suitable controls. 9 µl of the LAMP product was treated with the restriction enzyme- 1µl; NE buffer (×10) – 1 µl and incubated at 37°C for 15 min. The product was subjected to electrophoresis for 1 h and visualized under UV transilluminator and photographed using gel documentation system.

### LAMP amplification of clinical samples

The reaction mixture was prepared, and LAMP was run as described earlier, with DNA extracted described earlier and visualized under UV-transilluminator.

### Screening of clinical samples by PCR

All the 24 h broth culture, samples were screened by PCR for the presence of *cpa* gene as per the standard protocol [5].

### Comparison of diagnostic sensitivity of LAMP with PCR

Diagnostic sensitivity of LAMP was compared with PCR for detection of *C. perfringens* by testing 120 clinical samples by both the methods.

## Results and Discussion

**Figure-2:**
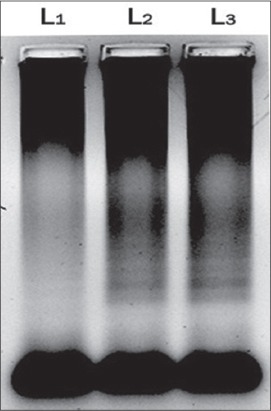
Agarose gel electrophoresis of amplicons of alpha toxin gene of *Clostridium perfringens* with different enzyme concentration. L1: 0.5 µl/25 µl of reaction mixture, L2: 1.0 µl/25 µl of reaction mixture, L3: 1.5 µl/25 µl of reaction of mixture.

**Figure-3:**
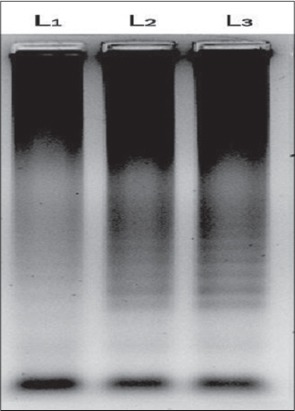
Agarose gel electrophoresis of amplicons alpha toxin gene of *Clostridium perfringens* with different primer concentration. L1: 10 pmol/µl (outer primers) and 40 pmol/µl (inner primer), L2: 10 pmol/µl (outer primers) and 80 pmol/µl (inner primers), L3: 10 pmol/µl (outer primers) and 160 pmol/µl (inner primers).

**Figure-4:**
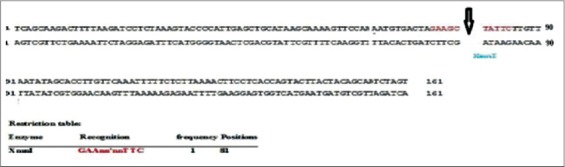
Restriction enzyme map for loop mediated isothermal amplification product of alpha toxin gene of *Clostridium perfringens* (BioEdit version 7.25 [12/11/2013] restriction mapping utility(c) 1998, Tom Hall, 162 base pairs).

**Figure-5:**
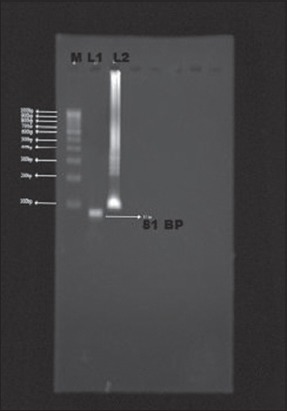
Agarose gel electrophoresis of alpha toxin gene of *Clostridium perfringens* with restriction enzyme (Xmnl) treatment. M: 100 bp DNA ladder, L1: 81 bp band (positive), L2: Loop mediated isothermal amplification ladder pattern (untreated).

The enzyme *Bsm* polymerase large fragment with high functional similarity to *Bst* DNApolymerase, the large fragment was used successfully in the present study in place of *Bst* DNApolymerase. The enzyme concentration of 1.0 µl was found to be optimum for LAMP reaction in the present study (Figure-2). Same concentration of the *Bst* DNA polymerase enzyme was used by earlier workers for successful LAMP reaction for amplification of *Bordetella pertussis*, Capri poxviruses viral genome [6,12,13]. The outer primers concentration of 10 pmol/µl and 160 pmol/µl of inner primers (FIP and BIP) were found to be optimum for LAMP reaction in the present study (Figure-3). Similar combinations of outer and inner primers were successfully used previously for detection of enterotoxigenic *C. perfringens* from meat samples [14]. The double strandard DNA in LAMP reaction mixture will be in dynamic equilibrium at the temperature around 65°C and one of the LAMP primers could anneal to the complimentary sequence to initiate LAMP reaction. Hence, heat denaturation of the double strandard DNA into a single strand, like with PCR was not recommended in LAMP reaction [5]. However, initial denaturation step of template DNA was added in the present study to increase the reaction efficiency as recommended by several previous workers [6,12,13,15-17]. To confirm that the amplification product had corresponding DNA sequences in the present investigation, the amplified product was digested with restriction enzyme *XmnI*. The enzyme produced single cut at 81 bp within specific recognition sequences in the LAMP amplified product of size 162 bp (Figure-4). The product size was visualized as single band (Figure-5), similar methods were adopted by previous authors to test the specificity of the LAMP products during the amplification of west nile virus and coronovirus genome [6,10,17]. Out of 120 samples screened by LAMP for alpha toxin specific primers 112 (93.30%) samples showed amplification of alpha toxin gene in the samples. Similarly, PCR using 24 h broth culture of fecal samples collected from lambs revealed amplification of alpha toxins gene in all LAMP positive samples indicating similar sensitivity of LAMP and PCR. A similar type of comparison between reverse transcription (RT)-LAMP and RT-PCR was made and it was shown that RT-LAMP was more sensitive than RT-PCR for detection of SARS corona virus from the clinical sample [17]. Though LAMP was said to be having advantages over PCR, the inability of multiplexing in LAMP for simultaneous amplification of more than one gene which is possible with multiplex PCR cannot be over ruled [18]. Moreover, PCR is a basic technique of DNA amplification with several variants and potential applications in molecular biology apart from its use as a simple diagnostic tool.

Hence, comparison of LAMP with PCR should be limited only to diagnostic applications in clinical laboratories in the developing counties.

Further, standardization of LAMP reaction for amplification of *etx* and *cpb* genes will help to identify the *C. perfringens* toxin types from the clinical samples. The test could be a suitable alternative to the PCR in detection of toxin types without help of sophisticated machinery like thermal cycler. Considering LAMPs simplicity in operation and high sensitivity, the test can be used for clinical diagnosis and surveillance of infectious diseases. To achieve these levels in resource-poor endemic areas, specimen processing methods, and a closed amplification and detection system need to be developed. This will facilitate the provision of a same-day testing strategy in even the most remote rural health facilities.

## Conclusion

Based on the present study we conclude that 24 h broth cultures of the fecal samples collected from enterotoxemia suspected lambs could be used for amplification of *C. perfringenscpa* gene by LAMP and PCR. Further the study reveals the efficacy of toxin genotyping from the clinical samples, compared with the conventional isolation, *in vitro* and *in vivo* toxin detection methods. Identification of the prevailing *C. perfringens* toxin types in the region will help to understand the epidemiology of enterotoxemia in sheep, particularly in lambs which helps in developing suitable control measures.

## Authors’ Contributions

BR is the student worked for MVSc thesis. NVK as a major guide designed and supervised the research work and prepared the manuscript. DS as a minor guide helped in work supervision and preparation of the manuscript.

## References

[1] Uzal F.A, Plumb J, Blackall L.L, O’Boyle D, Kelly W.R (1996). Detection of polymerase chain reaction of *Clostridium perfringens* producing epsilon toxin in feces and in gastrointestinal contents of goats. Lett. Appl. Microbiol.

[2] Dray T (2004). *Clostridium perfringens* type A and beta2 toxin associated with enterotoxaemia in a 5-week-old goat. Can. Vet. J.

[3] Khairy E.M, Dorgham S.M, Bakry M.A, Hakim A.S (2013). Molecular diversity of alpha toxin produced by *Clostridum perfringens* strains causing avian necrotic enteritis. World Appl. Sci. J.

[4] Meer R.R, Songer J.G (1997). Multiplex polymerase chain reaction assay for genotyping *Clostridium perfringens*. Am. J. Vet. Res.

[5] Vinod Kumar N, Sreenivasulu D, Reddy Y.N (2014). Prevalence of *Clostridium perfringens* toxin genotypes in enterotoxemia suspected sheep flocks of Andhra Pradesh. Vet. World.

[6] Parida M, Sannarangaiah S, Dash P.K, Rao P.V.L, Morita K (2008). Loop mediated isothermal amplification (LAMP):A new generation of innovative gene amplification technique;perspectives in clinical diagnosis of infectious diseases. Rev. Med. Virol.

[7] Notomi T, Okayama H, Masubuchi H, Yonekawa T, Watanabe K, Amino N, Hase T (2000). Loop mediated isothermal amplification of DNA. Nuc. Acids Res.

[8] Mori Y, Nagamine K, Tomita N, Notomi T (2001). Detection of loop mediated isothermal amplification reaction by turbidity derived from magnesium pyrophosphate formation. Biochem. Biophys. Res. Communication.

[9] Nagamine K, Watanabe K, Ohtsuka K, Hase T, Notomi T (2001). Loop mediated isothermal amplification reaction using a non denaturated template. Clin. Chem.

[10] Nagamine K, Hase T, Notomi T (2002). Accelerated reaction by loop mediated isothermal amplification using loop primers. Mol. Cell Probes.

[11] Sambrook J, Russel D.W (2001). Molecular Cloning.

[12] Kamachi K, Toyoizumi-Ajisaka H, Toda K, Soeung S.C, Sarath S, Nareth Y, Horiuchi Y, Kojima K, Takahashi M, Arakawa Y (2006). Development and evaluation of a loop mediated isothermal amplification method for rapid diagnosis of *Bordetella pertussis* infection. J. Clin. Microbiol.

[13] Das A, Babiuk S, Mclntosh M.T (2012). Development of a loop-mediated isothermal amplifiation assay for rapid detection of capri poxviruses. J. Clin. Microbiol.

[14] Kaneko K, Miyamoto K, Mimura K, Yumine N, Utsunomiya H, Akimoto S, McClane A.B (2011). Detection of enterotoxigenic *Clostridium perfringens* in meat samples by using molecular methods. Appl. Environ. Microbiol.

[15] Aryan E, Makvandi M, Farajzadeh A, Huygen K, Bifani P, Mousavi S, Fateh A, Jelodar A, Gouya M, Romano M (2010). A novel and more sensitive loop mediated isothermal amplification assay targeting IS6110 for detection of *Mycobacterium tuberculosis* complex. Microbiol. Res.

[16] Suzuki R, Ihira M, Enomoto Y, Yano H, Maruyama F, Emi N, Asano Y, Yoshikawa T (2010). Heat denaturation increases the sensitivity of the cytomegalovirus loop mediated isothermal amplification method. MicrobiolImmunol.

[17] Hong T.C, Mai Q.L, Cuong D.V, Parida M, Minekawa H, Notomi T, Hasebe F, Morita K (2004). Development and evaluation of a novel loop mediated isothermal amplification method for rapid detection of severe acute respiratory syndrome coronovirus. J. Clin. Microbiol.

[18] Hara Kudo Y, Yoshino M, Kojima T, Ikedo M (2005). Loop mediated isothermal amplification for the rapid detection of *Salmonella*. FEMS Microbiol. Lett.

